# A novel multimodal pharmacologic approach using guanfacine, N-acetylcysteine, and donepezil in severe TBI: a case series

**DOI:** 10.3389/fresc.2025.1648002

**Published:** 2025-10-24

**Authors:** Arman Fesharaki-Zadeh, Timothy Belliveau, Robert H. Pietrzak, Amy Arnsten

**Affiliations:** ^1^ Department of Neurology, Yale School of Medicine, New Haven, CT, United States; ^2^ Department of Psychiatry, Yale School of Medicine, New Haven, CT, United States; ^3^ Department of Social and Behavioral Sciences, Yale School of Medicine, New Haven, CT, United States; ^4^ Department of Neuroscience and Psychology, Yale School of Medicine, New Haven, CT, United States

**Keywords:** TBI, severe TBI, guanfacine, N-acetylcysteine, donepezil

## Abstract

Traumatic brain injury (TBI) remains a leading cause of long-term morbidity and disability worldwide. Individuals with moderate to severe TBI often experience persistent neurocognitive deficits, including short-term memory loss, executive dysfunction, and slowed cognitive processing for which there are currently no FDA-approved treatments. This case series investigates the synergistic use of guanfacine, N-acetylcysteine (NAC), and donepezil (GND) administered alongside ongoing cognitive rehabilitation, with treatment effects evaluated through pre- and post-intervention Montreal Cognitive Assessment (MoCA) scores. The guanfacine/NAC combination has previously been reported to improve working memory and executive function in individuals with mild TBI, suggesting its potential applicability to more severe TBI cases. Guanfacine, an alpha-2A agonist approved for ADHD, enhances prefrontal cortical function; Donepezil, a cholinesterase inhibitor, is widely used to treat cognitive symptoms in mild cognitive impairment and early dementia; and NAC, a potent antioxidant and glutamate modulator, has demonstrated neuroprotective effects across a range of clinical contexts, including TBI. Each of these agents has a well-established safety profile. The encouraging outcomes observed in this case series underscore the potential of the GND regimen as a multimodal pharmacologic approach to target the complex neurochemical disruptions following TBI. These preliminary findings warrant further investigation in larger, placebo-controlled trials in order to more rigorously assess the safety, efficacy, and translational potential of this intervention for mitigating chronic cognitive sequelae in individuals with moderate to severe TBI.

## Introduction

Traumatic brain injury (TBI) is an increasingly recognized global cause of morbidity and mortality (1). According to the Centers of Disease Control and Prevention, approximately 2.5 million TBI-related emergency department visits occur annually in the United States (2). In 2016, the estimated annual national cost of TBI-related care and management was $40.6 billion (3).

There is an increasing body of evidence linking TBI to higher risk of neurodegenerative disorders, including Alzheimer's disease (4), Parkinson disease (5), as well as chronic traumatic encephalopathy (6). Furthermore, TBI patients are at higher risk of developing psychiatric comorbidities, including depression (7), anxiety (8), impulsive behavior (9), suicidality (10) and psychotic symptoms (11).

Severe TBI (sTBI) can involve intracerebral hemorrhage or contusions, as well as axonal damage referred to as traumatic axonal injury (typically less than three lesions) vs. diffuse axonal injury (more than three lesions) depending on the severity of the impact (12). There are currently no Food and Drug Administration-approved medications for TBI (13). Hence, there is a great need for development of an effective pharmacotherapeutic regimen to address this treatment gap.

Cognitive impairments—particularly persistent memory deficits—are among the most frequently reported long-term consequences of TBI (14). The cortical cholinergic neurons and their ascending projections are especially vulnerable to TBI-induced biomechanical insult. Acetylcholine plays a key role in regulating arousal, attention and memory (15). A recent multicenter, double-blind, placebo-controlled, 10-week clinical trial demonstrated significant memory improvement in the donepezil treated group (16). The results of the study also supported a relatively safe and tolerable profile of donepezil.

The disruptions to the dopamine and noradrenaline networks due to TBI are fairly common (17). Damage to these networks has been linked to deficits in attention (18, 19), as well as learning and memory (20). Moreover, executive functions such as working memory, planning and inhibitory control, are commonly affected by TBI (21). Pharmacological agents such as methylphenidates have been shown to increase dopamine levels via inhibition of noradrenaline and dopamine transporters (22), and to increase dopamine release via D2-receptor modulation of vesicular trafficking (23). Other agents that modulate dopaminergic and noradrenergic activity include amantadine (24), dextroamphetamine (25), bromocriptine (26), atomoxetine (27), as well as levodopa (28), have been utilized in the treatment of moderate to severe TBI.

Our group has recently published findings on the use of guanfacine, an alpha-2A noradrenergic agonist, for treating patients with mild TBI who exhibit deficits in working memory and executive functioning (29). Guanfacine, marketed as Tenex (immediate release) and Intuniv (extended release), is an FDA-approved medication for attention-deficit hyperactivity disorder (ADHD). Preclinical studies have shown that guanfacine enhances prefrontal cortical (PFC) function by modulating cAMP–PKA–K⁺ signaling at post-synaptic alpha-2A receptors, thereby supporting and enhancing PFC neuronal firing and protecting dendritic spines from stress-related damage, and overall improvement in PFC functioning (30). Clinically, guanfacine leads to improvement in both executive function and working memory (31). In a study by McAllister et al. (32), guanfacine treatment was shown to enhance working memory in patients with mild TBI, as demonstrated by increased right PFC activation on fMRI.

NAC is a potent antioxidant which replenishes glutathione levels and has demonstrated mitochondrial protective effects (33). NAC also modulates the kynurenine pathway, reducing levels of kynurenic acid (KYNA), a neurotoxic metabolite that inhibits NMDA receptors (34). In our prior study, involving mild TBI patients, we have reported a significant neurocognitive benefit by the combined use of guanfacine and NAC, with proposed longitudinal antioxidant and anti-inflammatory benefits (29). The use of NAC has also been explored in larger clinical trials involving military members (35), as well as pediatric population (36). In the Hoffer et al. study, subjects receiving NAC within 24 h of the blast injury had an 86% chance of symptom resolution, including memory loss and neurocognitive dysfunction, with no reported side effects compared with 42% for those in the placebo group (35).

Donepezil, a cholinesterase inhibitor, is FDA-approved for the treatment for of mild cognitive impairment due to Alzheimer's disease (37). Prior TBI autopsy studies have reported significant but incomplete losses of basal forebrain cholinergic neurons and their projections in approximately 50% of sTBI cases (38). A recent multi-center trial by Arciniegas et al. (16) demonstrated that donepezil significantly improved persistent verbal memory impairment in individuals with predominantly sTBI during the chronic post injury period. In this trial, donepezil responders exhibited significant improvements in new learning, delayed recall, processing speed, and other cognitive domains, despite the study's limited sample size. Donepezil also has likely clinical benefits in attention, as a precursor function to verbal memory. It has a relatively favorable safety and tolerable profile.

**Figure 1:**
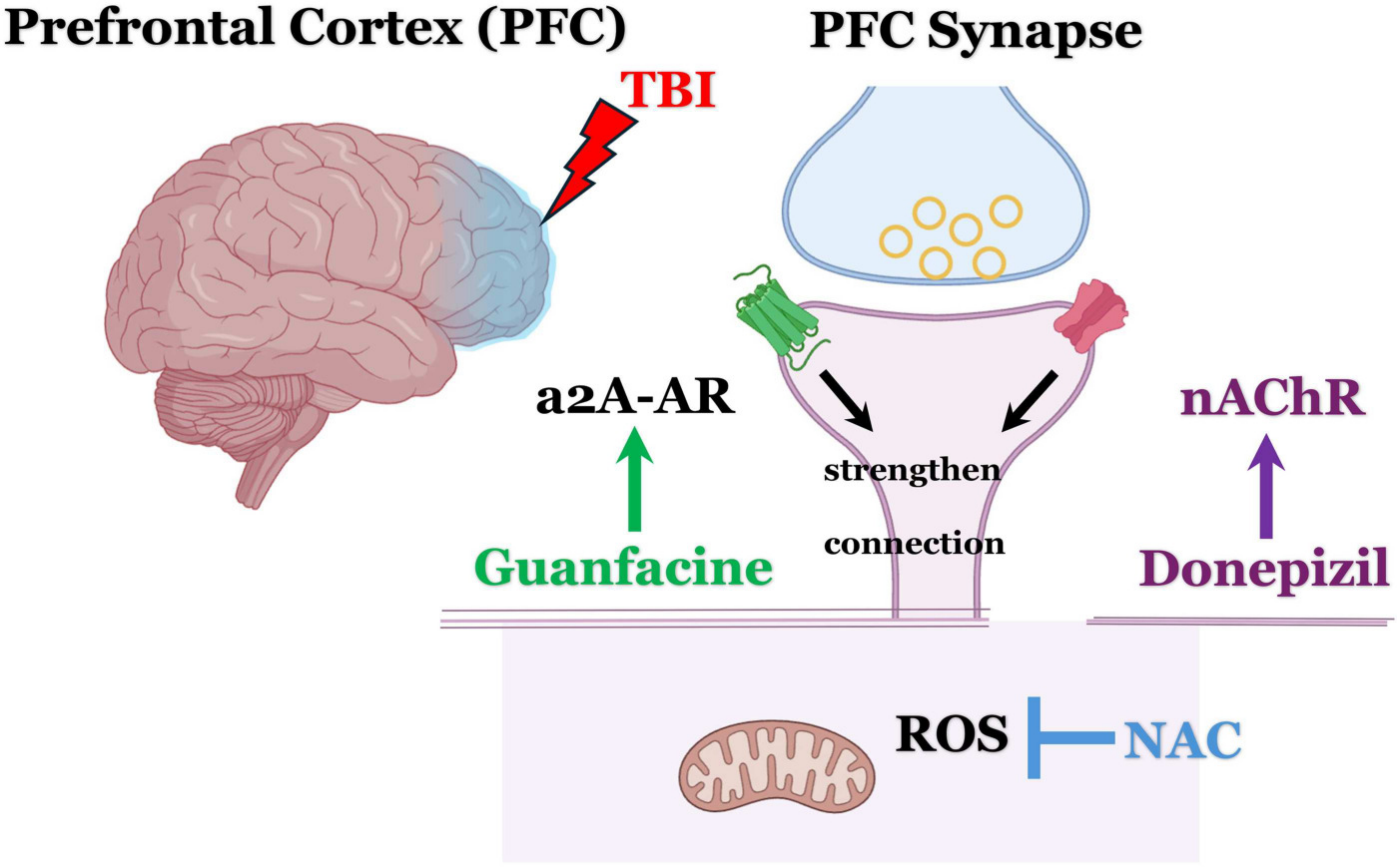
Schematic overview: mechanistic rationale for the use of guanfacine, N-acetylcysteine, and donepezil (GND) in treating neurocognitive impairment following severe TBI. Donepezil, increases the availability of acetylcholine (ACh), activates postsynaptic α7-nicotinic acetylcholine receptors (α7-nAChRs) and subsequently enhances NMDA receptor activity. This mechanism is thought to provide subthreshold benefits for working memory and executive functioning. Adding guanfacine likely offers a potentiating synergistic effect by activating α2A-adrenergic receptors (α2A-AR) and boosting postsynaptic signaling in the dorsolateral prefrontal cortex (DLPFC), particularly within Layer III pyramidal neurons. NAC provides mitochondrial protection via its robust anti-oxidant benefits and inhibiting the harmful effects of reactive oxygen species (ROS). Images created with BioRender.com.

Given the widespread neuroanatomical and neurocircuitry disruptions observed in severe traumatic brain injury (sTBI) and the associated global neurocognitive impairments—including deficits in working memory, executive functioning, and verbal and episodic memory—there is a strong rationale for the synergistic clinical benefits of combining guanfacine and donepezil. Furthermore, considering the persistent oxidative stress and neuroinflammatory processes that often follow sTBI, patients are also likely to benefit from the continued use of NAC (39). The proposed GND combination (guanfacine, NAC, donepezil) leverages distinct and complementary mechanisms of action, with minimal risk of drug–drug interactions, offering a comprehensive and well-tolerated therapeutic strategy for addressing the complex and chronic neurocognitive sequelae of sTBI (Figure 1).

The following two discussed cases were selected based on diagnosis of severe TBI, defined as Glascow Coma Scale (GCS) < 9, loss of consciousness >24 h and post-traumatic amnesia > 7 days (40). Mild and moderate TBI patients have not been included in the reported cases. Also, neither of the discussed patients were previously diagnosed with any neurodegenerative disorders prior to their injury.

## Case 1 (Mr. JP)

### Initial presentation

**Figure 2:**
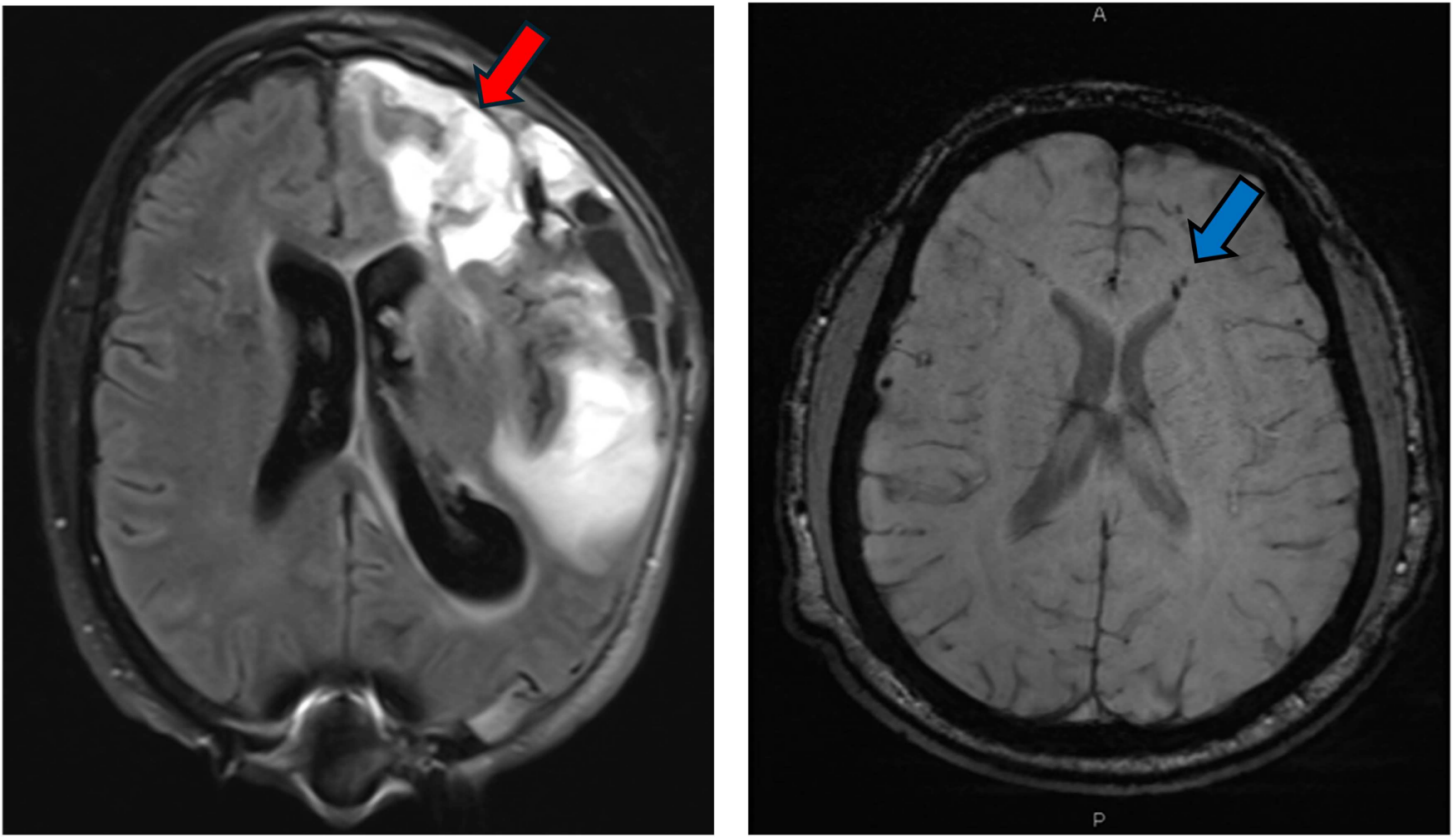
MRI FLAIR sequence demonstrating significant multicompartmental intraparenchymal hemorrhage (red arrow) post decompressive left hemicraniectomy for patient JP. MRI SWI sequence demonstrating multiple foci of intraparenchymal hemorrhages and diffuse axonal injury (DAI) for patient JS (blue arrow).

Mr. JP is a 60-year-old right-handed gentleman with a previous history of traumatic brain injury (age 52, left temporal intraparenchymal hemorrhage and left orbital roof fracture) who sustained a sTBI at age 59 following an unhelmeted motorcycle accident. His injuries were significant, including an extensive left frontotemporal hemorrhagic contusion, multicompartmental hemorrhages, subarachnoid hemorrhage, subdural hematoma, and a right sigmoid sinus thrombus (Figure 2). He also had abnormal EEGs with epileptiform abnormalities. His acute management involved decompressive hemicraniectomy, followed by cranioplasty in January 2020. He also sustained orthopedic injuries, including a right scapular fracture and left tibial and fibular fractures, requiring open reduction and internal fixation (ORIF).

### Follow up sessions

Following his hospitalization, Mr. JP completed extensive outpatient physical, occupational, and speech therapy and now receives ongoing home-based rehabilitation with nursing support. He was referred to our Concussion/TBI Clinic for neurocognitive evaluation due to persistent cognitive and functional deficits. His main complaints include short-term memory loss (forgetting conversations), word-finding difficulty, and expressive aphasia. His wife also reports impaired recall of familiar names and the need for assistance with medication management. He denies visuospatial problems.

Mr. JP remains on Keppra for seizure prophylaxis, with fair adherence (missed 1–2 doses weekly) and no reported seizures. He uses Alprazolam 1 mg PRN for episodic anxiety. While sleep disruption is not reported, his wife notes excessive daytime sleep. He endorses intermittent low mood without behavioral outbursts. He requires supervision for many IADLs and some ADLs, is not driving, but ambulates outdoors for light exercise. Amantadine 50 mg BID provides partial benefit but contributes to daytime sleepiness.

He was seen in the Concussion/TBI clinic after 8 months post injury. After initially being started on NAC regimen. He was subsequently started on donepezil (initiated at 5 mg nightly, titrated to 10 mg) for amnestic symptom. In a follow up session, Mr. JP was started on guanfacine ER (started at 1 mg nightly, titrated to 2 mg) after 13 months post initial injury, in order to address the persistent working memory and executive dysfunction. He also engaged in intensive outpatient speech/cognitive, physical, and occupational therapy after his initial visit.

## Case 2 (Mr. JS)

### Initial presentation

Mr. JS is a 30-year-old right-handed male with a longstanding history of medically refractory epilepsy, initially diagnosed at age 14, and a more recent history of sTBI sustained after being struck by a motor vehicle while riding a scooter His epilepsy history includes generalized tonic-clonic seizures (GTCs), with the first documented seizure occurring in 2008, involving convulsive activity, drooling, unresponsiveness, and postictal confusion. Despite initial treatment with Depakote and subsequent trials of Keppra XR, Topamax, and Tegretol, Mr. JS continued to experience seizures, which later evolved into predominantly nocturnal events characterized by leftward eye deviation, gasping sounds, facial twitching, limb stiffening, and postictal aphasia. Prior to his initial neurocognitive assessment, he was experiencing seizures two to three nights per week, though there were periods of remission. Mr. JS sustained a severe TBI. He reported being stationary at a red light with no memory of the collision, regaining awareness in the hospital. Acute injuries included a right-sided subarachnoid hemorrhage (Figure 2), multiple orthopedic fractures (pubic rami, patella, femur, sternum, ribs, and hand), and a prolonged ICU and rehabilitation course. Surgical interventions involved multiple orthopedic repairs and wound closures.

### Follow up sessions

**Figure 3:**
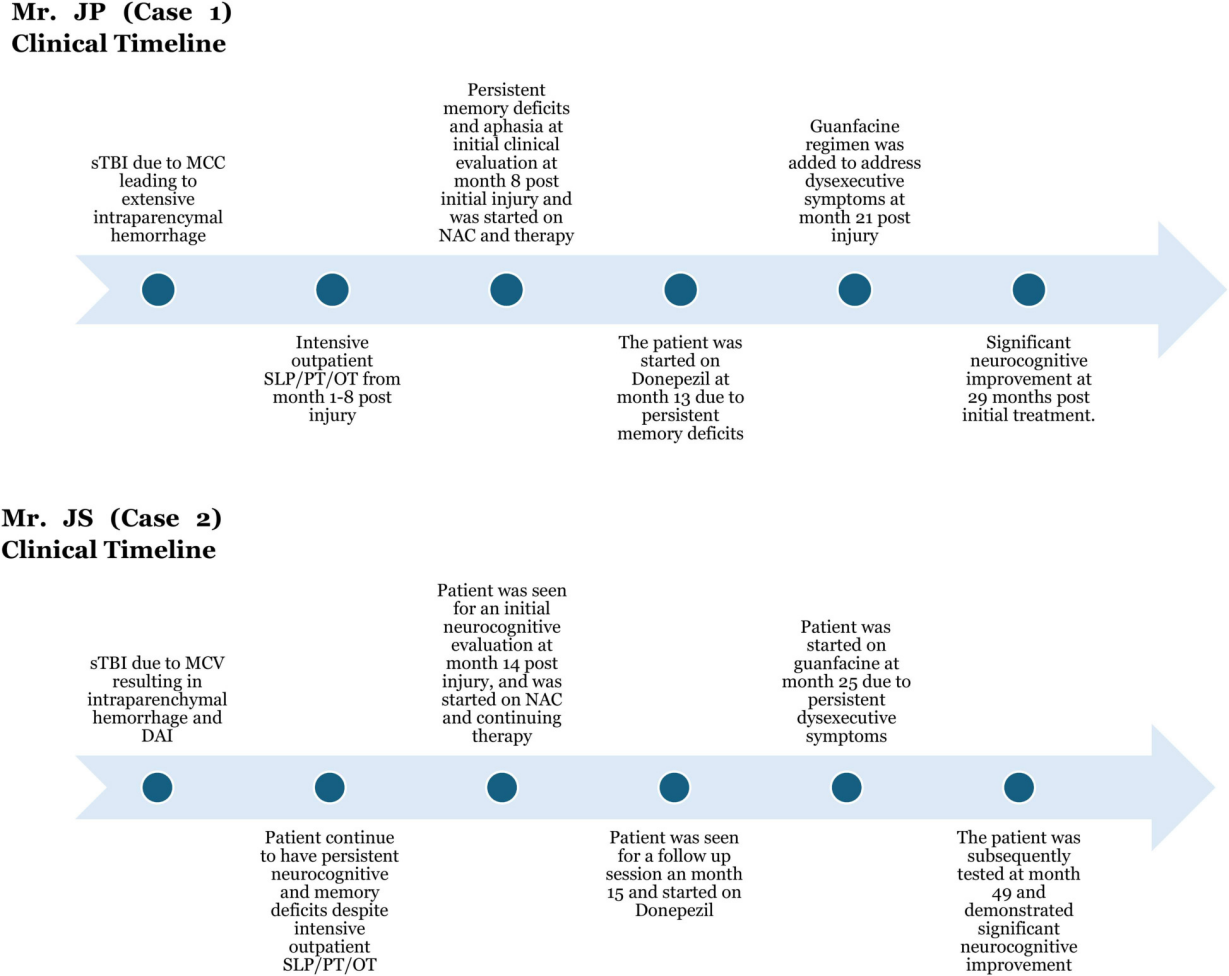
Detailed clinical timeline for case 1 and case 2. sTBI was the result of motorcycle collision (MCC) in case 1 and motor vehicle collision (MCV) in case 2. Intensive therapy included physical therapy (PT), occupational therapy (OT), and speech and language pathology (SLP) therapy. Both patients clinically benefited from the GND combination (guanfacine/N-acetylcysteine/donepezil).

Following rehabilitation, he used a walker initially, then transitioned to a cane, and is now ambulatory without assistive devices. Shortly after the initial visit at 14 months post injury the patient was started on NAC. Due to significant memory and recall deficits at 15 months post injury visit, Mr. JS was started on titrating dose of donepezil, with the target dose of 10 mg tab PO at night. As Mr. JS continued to have persistent working memory and executive functioning difficulties, he was started on guanfacine ER 1 mg tab PO at nighttime, which was titrated up to 2 mg after a one-month period at month 25 post injury. Mr. JS also underwent intensive outpatient speech/cognitive therapy in tandem with physical/occupational therapy. He was lost to follow for 1.5 years but was seen for a follow up session in the Concussion/TBI clinic. His subsequent assessment demonstrated significant global functioning improvement. JS's initial (T1: Time 1) MoCA assessment was at the time of his initial visit at 14 months post-injury, and his follow-up MoCA assessment (T2: Time 2) was at 4 years post-injury (Figure 3).

## Discussion

Patients with moderate to severe TBI frequently experience persistent neurocognitive deficits, including short-term memory loss, executive dysfunction, and slowed cognitive processing—symptoms for which there are currently no FDA-approved treatments. As such, clinicians often face significant challenges of treating these symptoms in the outpatient setting. The two cases described here represent a growing cohort of patients with moderate to severe TBI and enduring, debilitating neurocognitive deficits. The observed improvements following the use of GND combination therapy offer a potentially promising clinical approach for patients suffering from chronic post-traumatic encephalopathy.

In both Case 1 and Case 2, Montreal Cognitive Assessment (MoCA) scores showed robust and clinically meaningful improvements in nearly all cognitive domains (Table 1 and Figure 3). MoCA index scoring (41) was used to parse changes across the tested specific domains, including memory, language, visuospatial and executive functioning, with the most pronounced improvements noted on measures of memory and executive functioning.

**Table 1:** Montreal cognitive assessment (moCA) subscale scores for JP (case 1) and JS (case 2).

MoCA subscales	Case-1 Evaluation 1	Case-1 Evaluation 2	Case-2 Evaluation 1	Case 2 Evaluation 2
MoCA-TS	12	27	12	27
MoCA-MIS^a^	0	12	0	13
MoCA-EIS^a^	4	12	5	12
MoCA-VIS	4	7	4	5
MoCA-LIS	2	5	3	6
MoCA-AIS^a^	8	18	9	18
MoCA-OIS	5	6	4	6

Global improvement across multiple MoCA subscales post GND treatment. MoCA-TS, MoCA total score; MoCA-MIS, MoCA Memory Index Score; MoCA-EIS, MoCA Executive Index Score, MoCA Visuospatial Index Score; MoCA-LIS, MoCA Language Index Score; MoCA-AIS, MoCA Attention Index Score; MoCA-OIS, MoCA Orientation Index Score, with more detail description included here;

MoCA-TS: MoCA total score.

MoCA-MIS: MoCA Memory Index score defined as out of 15 score depending on recollection based on cueing.

MoCA-EIS: MoCA Executive Index Score calculated by adding raw scores for modified TMT-B. clock drawing, digits span forward and backward, letter A tapping, serial 7 subtraction, letter fluency and abstraction with scores ranging from 0 to 13.

MoCA-VIS: MoCA visuospatial index, defined as adding raw scores of the cube copy, clock drawing, and naming with score ranging from 0 to 7.

MoCA-LIS: MoCA language index score, defined as adding raw scores of naming, sentence repetition and letter fluency with score ranging from 0 to 6.

MoCA-AIS: MoCA attention index score, defined as adding raw scores for digit span forward and backward, letter A tapping, serial 7 subtraction, sentence repetition, words recall in both immediate recall trials with scores ranging from 0 to 18.

MoCA-OIS: MoCA orientation index score is the sum of points for the orientation section of the MoCA.

MoCA index scoring system is based on prior published work in MCI/AD population (41).

^a^ Additional statistical analysis included in Figure 3.

Our case series also demonstrates a novel multimodal pharmacological treatment strategy, and the utility of serial Montreal Cognitive Assessments to efficiently track cognitive changes after severe TBI. A recent study by Ratcliffe et al. (42), represents the most rigorous secondary analysis for test -retest stability of MoCA scores to date and include baseline (Time 1) and follow-up (Time 2) MoCA scores from the National Alzheimer's Coordinating Center Unform Data Set (NACC-UDS), for two clinical groups (dementia, mild cognitive impairment) and a normal control group. As found in many previous studies, interpretation of changes in MoCA scores are often influenced by ceiling effects, a highly negatively skewed distribution of scores, demographic differences, and test-retest stability coefficients, which are within the acceptable range for clinical groups but lower among normal/healthy control groups.

**Figure 4:**
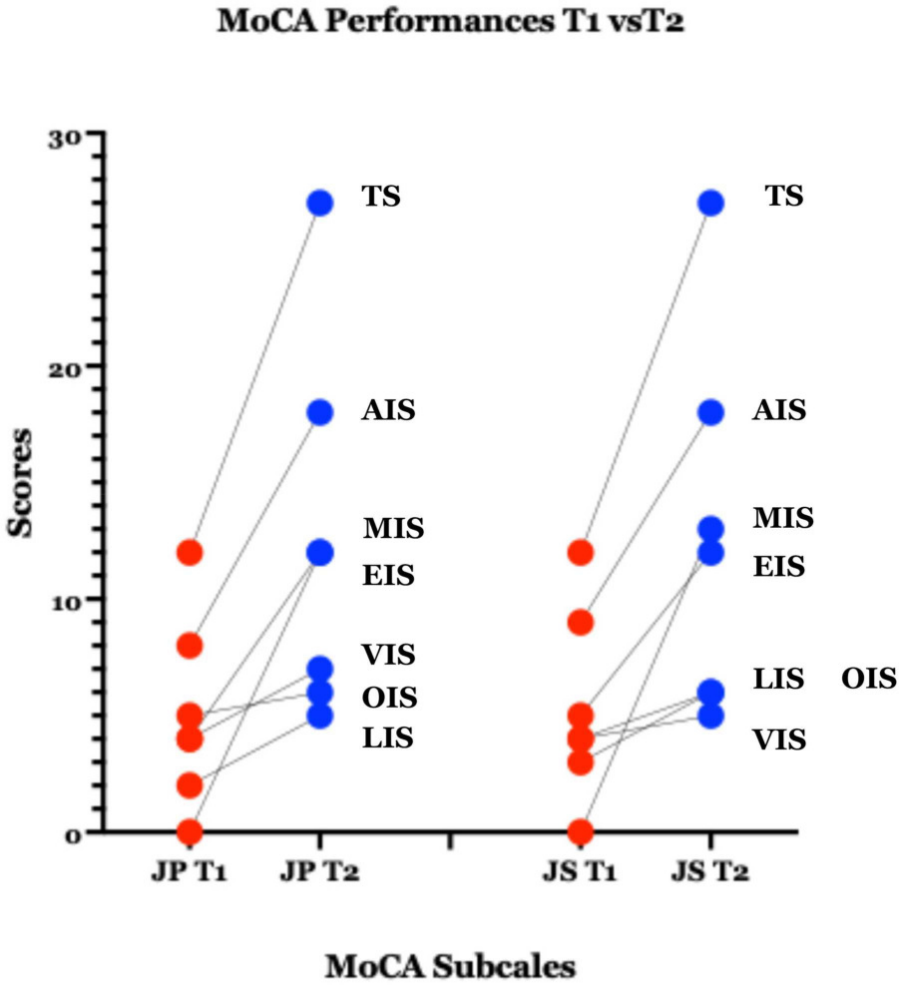
Graphic representation of the MoCA subscales, with each data point representing a MoCA Index Score, at Pre-treatment (T1:) and Post-treatment (T2) GND treatment; TS, total score; AIS, attention index score; MIS, memory index score; EIS, executive index score; VIS, visuospatial index score; OIS, orientation index score; LIS, language index score. *Paired-samples *t*-tests were conducted to examine changes in MoCA subscale scores from baseline to follow-up. Total MoCA scores were not analyzed, as both participants had identical scores at each time point (12 at baseline and 27 at follow-up), resulting in no within-pair variability. 1) MoCA-AIS: *t*(1) = 19.00, *p* = .033, Cohen's *d* = **13.48**, MoCA-MIS: *t*(1) = 25.00, *p* = .025, Cohen's *d* = **17.73**, MoCA-EIS**:** *t*(1) = 15.00, *p* = .042, Cohen's *d* = **10.64,** showed statistical significance. 2) MoCA-OIS: *t*(1) = 3.00, *p* = .205, Cohen's *d* = **2.13**, MoCA-VIS**:** *t*(1) = 2.00, *p* = .295, Cohen's *d* **=** **1.42,** did not reach statistical significance.

In an attempt to overcome the psychometric limitations associated with the traditional domain scores of the MoCA, we utilized a recently published method for the calculation of new Index scores (41). Both cases showed improvement in nearly all of the cognitive domains measured by the Index scores. More detailed statistical analysis using paired-samples t-test demonstrated significant improvement in MoCA-AIS, MoCA-MIS and MoCA-EIS subscales, while this did not apply to the MoCA-OIS and MoCA-VIS subscales (Figure 4).

The GND combination therapy—comprising guanfacine, NAC, and donepezil—was well-tolerated and leveraged the independent FDA-approved safety profiles of each agent: guanfacine for ADD/ADHD (43), NAC for acetaminophen toxicity (44) and donepezil for mild cognitive impairment (45). Given the multifaceted nature of cognitive impairment in sTBI, including amnestic, working memory, and executive function deficits, a multimodal treatment approach targeting distinct neurochemical systems (i.e., cholinergic and noradrenergic) is warranted.

The role of cognitive and speech therapy is also critical and should be highlighted. Both patients underwent a rigorous outpatient cognitive rehabilitation and reported significant subjective benefits. The essential role of cognitive rehabilitation for TBI and stroke patients is well-established (46–48). We posit that the integration of pharmacological treatment with rehabilitation may yield synergistic effects, accelerating cognitive recovery and functional gains (49–51). Of note, donepezil has also been shown to reduce apathy in dementia and stroke patients, (52, 53). It is entirely conceivable that the use of donepezil in GND combination regimen led to further engagement, motivation and participation in rehabilitation.

A recent multicenter randomized controlled trial by Arciniegas et al. (16), found that donepezil led to significant improvement in verbal learning measured by Hopkins Verbal Learning Test (HVLT), as well as delayed recall and processing speed vs. placebo. However, the study did not find significant effects on working memory or executive functioning. In contrast, our case reports suggest measurable improvements across these domains as well. Mechanistically, it is plausible that donepezil may enhance memory retrieval and processing speed by increasing acetylcholine availability and stimulating α7 nicotinic acetylcholine receptors (α7-nAChRs) and NMDA receptors (54, 55) (Figure 1), which arguably would have “subthreshold” benefits for working memory/executive functioning. Adding guanfacine likely provides a potentiating synergistic benefit of activating the a2A-adrenergic receptors and increasing the postsynaptic signal in DLFPC Layer III pyramidal neurons. Given the extent of DLPFC damage often seen in sTBI, neither agent alone may be sufficient, but their combination likely yields a synergistic therapeutic effect.

Memory formation could be conceptualized in three stages: encoding, consolidation, and retrieval (56). Donepezil has demonstrated benefits in retrieval and processing speed (16), while the addition of guanfacine may enhance encoding and executive functioning (29, 43) in the two reported cases. For sTBI patients who often suffer from global cognitive deficits, like the patients in our cases, GND combination treatment offers the advantage of addressing multiple stages of memory processes. NAC monotherapy has been explored in TBI and has shown clinical efficacy in several published studies (35, 57, 58). The addition of NAC, which has demonstrated efficacy in prior TBI studies, may offer neuroprotection through its antioxidant and anti-inflammatory properties, addressing the chronic neuroinflammatory state often seen post-TBI (59, 60).

## Limitations

The limitation of the study includes a limited sample size, the absence of a control group with no pharmacological treatment, placebo, or an alternative pharmacological regimen, and the absence of female patients in this case series. Considering the promising cognitive outcomes anecdotally observed in a small series of our clinic patients, we anticipate future studies with a larger and more diverse TBI sample, for which we will also have a broader set of neuropsychological test data to compare with MoCA scores. However, given the success of the treatment of these two cases, the GND treatment combination has been used in additional patients in our clinical setting. It is important to note that the reported cases series does not report on a potential control group without the GND combination treatment. It also does not provide the direct group comparison with other treatment groups, including patients that received guanfacine/NAC or donepezil/NAC only combinations.

Another potential limitation is not including female patients in this case series, which needs to be addressed in future studies for greater translational value. An additional limitation of the study includes lack of detailed neuropsychological assessment prior and post combination treatment therapy. Although both patients were able to complete the baseline neuropsychological assessments, neither were able to complete the post treatment neuropsychological assessment, mainly due to insurance and cost related issues. Although the MoCA score improvements in both patients were robust, it will be more clinically translatable to replicate these results based on comprehensive pre- and post- GND treatment neuropsychological assessment in future studies.

## Conclusion

Chronic neurocognitive impairments following moderate to severe TBI remain among the most disabling and treatment-resistant sequelae of brain injury. Given the lack of currently FDA-pharmacological treatments for sTBI patients, there is a critical unmet need for effective therapeutic strategies. This case series highlights the potential utility of a multimodal therapeutic approach combining guanfacine, N-acetylcysteine, and donepezil (GND), alongside structured cognitive rehabilitation. Larger, controlled studies are needed to validate these findings and assess their broader clinical applicability.

## Data Availability

The datasets presented in this article are not readily available because of ethical and privacy restrictions. Requests to access the datasets should be directed to the corresponding author.
